# Moral Injury, Chaplaincy and Mental Health Provider Approaches to Treatment: A Scoping Review

**DOI:** 10.1007/s10943-022-01534-4

**Published:** 2022-03-15

**Authors:** Kimberley A. Jones, Isabella Freijah, Lindsay Carey, R. Nicholas Carleton, Peter Devenish-Meares, Lisa Dell, Sara Rodrigues, Kelsey Madden, Lucinda Johnson, Fardous Hosseiny, Andrea J. Phelps

**Affiliations:** 1 Phoenix Australia - Centre for Posttraumatic Mental Health, Department of Psychiatry, University of Melbourne, Carlton, VIC 3053 Australia; 2 Department of Public Health, La Trobe University, Melbourne, Australia; 3 Department of Psychology, University of Regina, Regina, Canada; 4 Graduate School of Business, Queensland University of Technology, Brisbane, Australia; 5 Canadian Centre of Excellence on Post-Traumatic Stress Disorder (PTSD) and Related Mental Health Conditions, Ottawa, Canada

**Keywords:** Moral injury, Treatment, Chaplaincy, Mental health practitioner, Psychological, Spiritual, Review

## Abstract

The aim of this research was to describe the evidence examining the approaches taken by mental health providers (MHPs) and chaplains to address symptoms related to moral injury (MI) or exposure to potentially morally injurious events (PMIEs). This research also considers the implications for a holistic approach to address symptoms related to MI that combines mental health and chaplaincy work. A scoping review of literature was conducted using Medline, PsycINFO, Embase, Central Register of Controlled Trials, Proquest, Philosphers Index, CINAHL, SocINDEX, Academic Search Complete, Web of Science and Scopus databases using search terms related to MI and chaplaincy approaches or psychological approaches to MI. The search identified 35 eligible studies: 26 quantitative studies and nine qualitative studies. Most quantitative studies (*n* = 33) were conducted in military samples. The studies examined interventions delivered by chaplains (*n* = 5), MHPs (*n* = 23) and combined approaches (*n* = 7). Most studies used symptoms of post-traumatic stress disorder (PTSD) and/or depression as primary outcomes. Various approaches to addressing MI have been reported in the literature, including MHP, chaplaincy and combined approaches, however, there is currently limited evidence to support the effectiveness of any approach. There is a need for high quality empirical studies assessing the effectiveness of interventions designed to address MI-related symptoms. Outcome measures should include the breadth of psychosocial and spiritual impacts of MI if we are to establish the benefits of MHP and chaplaincy approaches and the potential incremental value of combining both approaches into a holistic model of care.

## Introduction

Moral injury (MI) refers to the enduring psychosocial and spiritual harms that can result from exposure to situations or events that occur in high stakes situations and involve transgression of one’s deeply held moral convictions of right and wrong, or perceived betrayal by those in positions of authority. (Litz et al., 2009; Shay, 2014). These events are described as potentially morally injurious events (PMIEs), recognizing that not everyone exposed will be impacted in the same way. Evidence from a range of studies in United States (US) military samples suggests that individuals exposed to PMIEs are at greater risk of developing mental health issues, including more severe post-traumatic stress disorder (PTSD), depression, anxiety, and suicidality (summarized in Koenig & Al Zaben, 2021).

Although symptoms often co-occur and can overlap with PTSD, MI appears to be functionally distinct from PTSD (Farnsworth et al., 2017; Shay, 2014). Studies conducted with active military personnel have specifically distinguished MI symptom profiles from PTSD with respect to guilt, self-blame, and shame, rather than gaps in memory of trauma, flashbacks, and nightmares (Bryan et al., 2018; Litz et al., 2018). There may also be different causal mechanisms involved in MI and PTSD at the neurophysiological level (Barnes et al., 2019).

There has been a surge of interest in MI research in recent years focusing on efforts to understand and frame the construct of MI and the prevalence of MI in diverse populations (e.g., military personnel, veterans, police and other first responders), recently including healthcare workers in the context of the COVID-19 pandemic (Koenig & Al Zaben, 2021). The research has been led by mental health-related disciplines (e.g., psychology, psychiatry) as well as spiritual and religious disciplines (e.g., chaplaincy), all of which have played important roles in conceptualizing and developing approaches to address MI. Standard interventions for PTSD, such as Prolonged Exposure (PE), may be helpful for addressing some of the psychological distress associated with exposure to PMIEs (e.g., Paul et al., 2014; Wachen et al., 2016); nevertheless, new intervention approaches directly targeting the specific causes and consequences of MI may also benefit some patients (e.g., Gray et al., 2012; Maguen et al., 2017). There is evidence that spiritually integrated psychotherapies, as well as chaplain facilitated pastoral care approaches, may be effective adjuncts or alternatives for addressing MI (e.g., Carey & Hodgson, 2018; Cenkner et al., 2021). The potential benefits of these approaches may be associated with addressing the spiritual impacts of moral transgressions (Carey et al., 2016; Koenig & Al Zaben, 2021).

Despite the proliferation of research into MI as a construct, there is a dearth of literature examining the effectiveness of MHP or chaplaincy intervention approaches. An early integrated narrative review of MI interventions by Griffin et al. (2019) identified studies using: (1) extant and adaptations of extant interventions for PTSD (i.e., Prolonged Exposure [PE], Cognitive Processing Therapy [CPT], Spiritually Integrated CPT); (2) alternative and adjunctive interventions for MI (i.e., Acceptance and Commitment Therapy, Adaptive Disclosure, Impact of Killing); and (3) interventions integrating spirituality (i.e., Building Spiritual Strength). The review identified some evidence for Adaptive Disclosure and Impact of Killing in addressing MI, but most of the intervention approaches were not associated with efficacy or effectiveness trials (Griffin et al., 2019). Since that time, the evidence base has increased to include several intervention trials, including trials of relatively novel interventions.

A recent narrative review by Harris et al. (2021) identified eight spiritually integrated interventions for PTSD and MI, including an updated summary of the available evidence base. While this review included three interventions that target MI (Adaptive Disclosure, Impact of Killing and Building Spiritual Strength) other intervention approaches used by mental health professionals and chaplains to address MI were not included. A second narrative review (Koenig & Al Zaben, 2021) identified several additional psychological approaches, spiritual/religious integrated approaches and pastoral care approaches. This review provided a brief description of the available approaches to MI but did not present evidence of intervention effectiveness.

Thus, despite these recent reviews, there appears to be a gap in the MI literature that can be addressed by a review employing a systematic methodology to identify the currently available psychological, chaplaincy, and integrated approaches to addressing MI, alongside a systematic summary of evidence of effectiveness. The current scoping review was designed to systematically identify and map the approaches taken by MHPs and chaplains to address moral injury, including any evidence of effectiveness.

## Method

A scoping review was conducted involving a systematic search of studies examining MHP and/or chaplaincy approaches to support adults who have experienced MI or been exposed to PMIEs (Arksey & O’Malley, 2005). The review adhered to the PRISMA Extension for Scoping Reviews: Checklist and Explanation (PRISMA-ScR) statement (Tricco et al., 2018).

### Identifying Relevant Studies

Searches were conducted on 11 electronic databases (Medline, PsycINFO, Embase, Central Register of Controlled Trials, Proquest, Philosphers Index, CINHAL, SocINDEX, Academic Search Complete, Web of Science and Scopus) combining terms related to MI and chaplaincy approaches or psychological approaches to MI (See Appendix 1 for search strategy). Manual searches of the reference lists of key relevant studies were conducted to identify any additional relevant publications. The search strategy included all publication types except for conference abstracts, published from database inception until August 2021.

### Study Inclusion and Exclusion Criteria

Eligible studies included any research designs that reported on either MHP approaches or chaplaincy approaches to support adults reporting MI symptoms or exposure to a PMIE. The research designs included case studies, pilot studies, or trials of interventions reporting on intervention efficacy, as well as qualitative descriptive studies or qualitative evaluations of interventions. Studies were restricted to English language, peer-review journals, and unpublished dissertations. The search excluded conceptual studies that reported on untested recommendations or interventions to address MI, protocols of ongoing clinical trials, or interventions that were not delivered by MHPs or chaplains.

Following a pilot test of eligibility criteria, records were initially screened on title and abstract by one reviewer (I.F.), with 20% of studies screened by a second reviewer (K.J.). All records not excluded on the basis of title and abstract were passed on for full-text review. Two reviewers (I.F. and K.J.) independently reviewed full-text records for potentially eligible studies. Any disagreements at the full-text screening stage were resolved by discussion, or through adjudication with a third reviewer (A.P.). Records deemed ineligible at full-text screening were excluded with the reason recorded. All screening was conducted using the systematic review management tool Covidence (Covidence Systematic Review Software).

### Data Charting

Data were charted using a standardized data collection form by a single reviewer, capturing information on key study characteristics. The data collection form included author, year, title, study setting, study design, population, sample size, data collection methods, intervention characteristics (e.g., intervention name, duration, number of sessions, session details), outcomes, and important findings. The intent of this scoping review was to ascertain the size and scope of the available literature. No appraisal was conducted of evidence quality.

### Summarizing and Reporting Findings

Results were summarized using a narrative descriptive synthesis approach (Khalil et al., 2016) with evidence from quantitative and qualitative studies categorized according to the facilitator (i.e., MHPs, chaplains, and combination of MHPs and chaplains) and were supported by a table of key study characteristics. Summaries related to study setting and population, psychological or chaplaincy approaches, along with outcomes assessed and broad key findings.

## Results

### Search Results

Electronic searches yielded 14,986 records, with 5705 of these (minus duplicates) screened on the basis of title and abstract. Of these, 196 were subject to full-text review, with 31 studies deemed eligible for inclusion in the current scoping review. Six additional records were identified through manual citation searches, four of which were deemed eligible for inclusion. Overall, 35 studies (*n* = 26 quantitative; *n* = 9 qualitative) were included in the review (see Table 1).

**Table 1:** Key characteristics of included studies (*k* = 35)

Author *Country*	Study setting	Study population	Design	Intervention	Key findings
*Mental health practitioner delivered interventions*					
Bluett (2017) *US*	VAMC	33 Veterans (88% male) with PTSD who completed an EBP for PTSD	Pre/post	*ACT* 8 weekly group sessions (120 min) *Adaptation of a pre-existing ACT manual developed for adult individuals with PTSD removing trauma-processing components*	• 64.7% of veterans showed a favorable response to treatment (5-point reduction in PTSD symptoms) • Significant improvement in PTSD symptoms, wellbeing, and depression, with no differences found for shame and quality of life Baseline PCL: *M* = 45.07 (SD = 15.27) Post-treatment PCL: *M* = 40.52 (SD = 14.02) • Significant improvement in MI events by perceived transgressions of MI from baseline (*M* = 17.23, SD = 6.27) to post-treatment (*M* = 14.86, SD = 6.77, *p* = .033) However these were not sustained, with a significant increase in scores at follow-up (*M* = 19.77, SD = 6.63, *p* = .006) • Non-significant reduction in number of reported MI events by perceived betrayals from baseline (*M* = 7.94, SD = 2.76) to post-treatment (M = 8.10, SD = 2.50, *p* = .94), with a significant increase in scores at follow-up (*M* = 9.33, SD = 4.10, *p* = .006)
Borges (2019) *US*	VAMC	1 Male veteran with PTSD, suicide ideation and exposure to PMIEs	Case study	*ACT for moral injury* 12 weekly individual telehealth sessions (90 min) *Transdiagnostic treatment focused on acceptance of moral pain using values rather than challenging the moral pain. This includes value clarification, mindfulness, emotion-regulation, distress tolerance skills*	• The veteran reported enhanced motivation for treatment-seeking and a reduction in trauma symptoms through goal envisioning, narrative reconstruction, neuro-coupling, self-transcendence, and externalization • Slight improvement in PTSD symptoms and depression at post-treatment Baseline PCL: 61 Post-treatment PCL: 59 Baseline PHQ: 21 Post-treatment PHQ: 19 •Slight improvement in MI as an outcome Baseline CFQ-MI: 44 Post-treatment CFQ-MI: 37 Baseline EMIS: 65 Post-treatment EMIS: 64
Farnsworth et al. (2017) *US*	VAMC	11 Male veterans in a PTSD residential treatment program completing group ACT (for MI) over a period of 2 weeks	Qualitative (interviews)	*ACT* 6 group sessions (75 min) over 2 weeks *A transdiagnostic approach that helps patients respond to challenging events and create a meaningful life by focusing on acceptance, mindfulness, value clarification and behavioral commitment. To align with the policies of the residential program, there were no explicit discussions of MIEs*	• All veterans reported that they benefited from the intervention, particularly from the distinct clinical elements that are hallmark of ACT (e.g., diffusion—developing a new relationship with their thoughts; reconnect with values) • Veterans reported that they would recommend it to other veterans • Some veterans noted that 6 sessions were too short, with others reporting difficulty in completing the treatment concurrently to other interventions as part of the residential program
Gray et al. (2012) *US*	Marine Corps base camp	44 Active-duty marines and Navy Corps personnel (95% male) with PTSD	Open trial	*Adaptive disclosure* 6 weekly individual sessions (90 min) *An emotion-focused psychotherapy developed specifically for AD personnel to address MI, traumatic loss/grief and impacts of life-threatening experiences. Sessions focus on identifying the index event, psychoeducation, imaginal exposure, and experiential processing*	• Significant improvements were found for PTSD, depression symptoms, post-traumatic cognitions, and post-traumatic growth at post-treatment Baseline PCL: *M* = 60.13 Post-treatment PCL: *M* = 50.55 Baseline PHQ: *M* = 14.32 Post-treatment PHQ: *M* = 10.97 Baseline PTCI: *M* = 10.95 Post-treatment PTCI: *M* = 9.48 Baseline PTGI: *M* = 2.28 Post-treatment PTGI: *M* = 2.58 • A non-significant improvement was found for alcohol use Baseline AUDIT: *M* = 7.80 Post-treatment AUDIT: *M* = 6.49 • Participants reported satisfaction with the treatment
Litz et al. (2021) *US*	Military garrison	122 Active-duty personnel (92% male) with PTSD	RCT	*Adaptive disclosure* 8 weekly individual sessions (90 min) See Gray et al. (2012) Comparator: CPT Cognitive version 12 weekly individual sessions (60 min) *CPT without the written account of the trauma*	• AD was found to be non-inferior to CPT-C, with no difference found between CAPS total severity change score between AD and CPT-C • AD Baseline CAPS: *M* = 74.58 (SD = 19.25) Post-treatment CAPS: *M* = 56.89 (SD = 28.12) Baseline PHQ-9: *M* = 15.42 (SD = 5.86) Post-treatment PHQ-9: *M* = 12.90 (SD = 6.56) Improved or recovered: 24% Dropout: 37% • CPT-C Baseline CAPS: *M* = 76.53 (SD = 18.43) Post-treatment CAPS: *M* = 53.33 (SD = 31.68) Baseline PHQ-9: *M* = 16.16 (SD = 7.11) Post-treatment PHQ-9: *M* = 13.39 (SD = 8.10) Improved or recovered: 25% Dropout: 40%
de la Rie et al. (2021) *NL*	Dutch mental health institute	1 Refugee male (who served in the military in his country of origin) with PTSD	Case study	*Brief eclectic psychotherapy for moral trauma* 16 weekly individual sessions *Comprises cognitive- behavioral, psychodynamic, constructivist, and systemic psychotherapy components, including psychoeducation, imagery exposure, letter writing, finding meaning and activation*	• Improvements were found for PTSD and MI symptoms at post-treatment Baseline CAPS: 25 Post-treatment CAPS: 19 Baseline PCL-5: 49 Post-treatment PCL-5: 36 Baseline MIAS: 31 Post-treatment MIAS: 28
Held et al. (2021) *US*	Outpatient mental healthcare center	150 Veterans and 11 active-duty personnel (91% male) with PTSD	Pre/post	*Massed CPT (for PTSD)* 14 daily individual session (50 min) 13 daily group sessions over 3 weeks (120 min) Daily mindfulness sessions, yoga sessions, psychoeducation and case management *Cognitive-behavioral treatment for PTSD that addresses safety, trust, power, control, self-esteem, and intimacy through identifying, challenging, and replacing unhelpful thoughts/beliefs and a written account of the traumatic experience* Note. CPT was not modified for individuals exposed to PMIEs	• 80% of veterans endorsed committing or witnessing transgressions consistent with MI • Large reductions in PTSD and depression symptoms • PTSD and depression symptom improvement from baseline to post-treatment did not differ based on MI history or index trauma type and did not predict changes in symptom outcomes • No differences were found between groups in the number of individuals who achieved probable remission, suggesting that military personnel exposed to PMIEs can be effectively treated with massed-CPT
Hawkins (2021) *US*	Army base	155 Active-duty personnel (91% male) with PTSD who completed group or individual CPT-C	RCT (secondary analysis)	*CPT—Cognitive version* (for PTSD) 12 biweekly group (90 min) or individual (60 min) sessions *Cognitive-behavioral treatment for PTSD that addresses safety, trust, power, control, self-esteem, and intimacy through identifying, challenging, and replacing unhelpful thoughts/beliefs. This version of CPT does not include the written account of the trauma*	• Participants reported reductions in PTSD severity, 72% of military personnel meet the criteria for PTSD at post-treatment • No significant differences were found in PTSD, depression, anxiety, or suicide ideation between individuals who endorsed a moral injury index trauma compared to those with other trauma types (e.g., life-threatening or traumatic loss) • Moral injury trauma (*n* = 60) Baseline PCL: *M* = 54.4 (SD = 10.8) Post-treatment PCL: *M* = 45 (SD = 15.2) Baseline PHQ: *M* = 28.7 (SD = 11.1) Post-treatment PHQ: *M* = 20 (SD = 13.3) • Life threat trauma (*n* = 64) Baseline PCL: *M* = 54.2 (SD = 9.9) Post-treatment PCL: *M* = 42 (SD = 16.4) Baseline PHQ: *M* = 28.1 (SD = 11.1) Post-treatment PHQ: *M* = 20 (SD = 14.7) • Traumatic loss trauma (*n* = 31) Baseline PCL: *M* = 54.4 (SD = 12.4) Post-treatment PCL: *M* = 46.2 (SD = 14.7) Baseline PHQ: *M* = 27.1 (SD = 11.3) Post-treatment PHQ: *M* = 26.2 (SD = 14.7) Regardless of trauma type, individuals completing the individual mode of CPT-C reported better mental health outcomes than those completing group CPT-C
Pearce et al. (2018) *US*	VAMC	1 Veteran with PTSD and exposure to PMIEs	Case study	*Spiritually Integrated—CPT* 12 weekly or biweekly individual sessions (50-60 min) *Individualized psychotherapy focusing on correcting erroneous interpretations through gradual exposure, processing and cognitive restructuring using spiritual resources (e.g., mercy, repentance, forgiveness, spiritual surrender, prayer/contemplation, divine justice, hope, and divine affirmations), and rituals (e.g., confession, penance), adapted to the individual's specific beliefs (e.g., Christian, Jewish, Muslim, Buddhist, and Hindu)*	• Improvements were found in PTSD symptoms at post-treatment Baseline PCL: 57 Post-treatment PCL: 31
Murray and Ehlers (2021) *UK*	Hospital	1 Female doctor exposed to PMIEs	Case study	*Cognitive therapy for PTSD *(addressing MI) 12 weekly individual sessions (90 min) *Ehlers and Clark's (2000) cognitive model of PTSD adapted to include MI-related content*	• Improvements were found in PTSD, depression and functioning at post-treatment Baseline CAPS: 34 Post-treatment CAPS: 2 Baseline PCL: 44 Post-treatment PCL: 0 Baseline BDI-II: 23 Post-treatment BDI-II: 1 Baseline WSAS: 19 Post-treatment WSAS: 2
Borges et al. (2020) *US*	VAMC	14 Male veterans who completed an EBP for PTSD and exposure to PMIEs	Qualitative (interviews)	*EBPs for PTSD* (CPT, PE)	Four key themes emerged: • MI was not identified and not discussed enough during therapy • Therapeutic relationships can either facilitate or inhibit the discussion of MI • PE and CPT have limited impact on MI symptoms • It is difficult for veterans to cope with MI following treatment
Held et al. (2018) *US*	Outpatient mental healthcare center	2 Male veterans with PTSD and exposure to PMIEs	Case study	*Cognitive processing therapy or prolonged exposure* (for PTSD) See Paul et al. (2014) and Held et al. (2021)	• Improvements in PTSD and depression symptoms at post treatment in both cases • Case #1—PE Baseline CAPS: 55 Post-treatment CAPS: 25 • Case #2—CPT Baseline CAPS: 65 Post-treatment CAPS: 13
Burkman et al. (2019) *US*	VAMC	10 MHPs (70% female) working with veterans with PTSD or comorbid PTSD and AUD were given the IOK materials to assess	Qualitative (interviews)	*Impact of killing* See Maguen et al. (2017)	• IOK treatment was found to be acceptable and fulfilled a clinical need not met by other evidence‐based treatments for PTSD • Treatment structure allows for therapist’s flexibility but may be too short in length to achieve aims • Regarding content, some MHPs reported that they would like further training on the stigmatizing topics, but agreed that IOK targets novel concepts (i.e., focus on morality and spirituality, sessions on forgiveness and making amends, and acceptance of guilt) • Noted that it could be generalizable to other moral injurious acts (e.g., police officers who have harmed others in the course of duty)
Maguen et al. (2017) *US*	VHA (outpatient clinic, hospitals), Vet centers	35 Male veterans with PTSD who endorsed distress from killing or being responsible for the death of another in a war zone	Pilot RCT	*Impact of killing* 6–8 weekly individual sessions (60-90 min) *A CBT intervention designed to be adjunctive to TF-CBT (e.g., PE, CPT). Has explicit focus on the act of killing and acknowledges that the killing may have crossed personal/shared morals thereby causing MI. Focus on self-forgiveness and making amends* Note. Does not have an emphasis on the spiritual dimensions of MI Comparator: Waitlist	• In comparison to the control group, significant improvements were found for PTSD symptoms, general psychiatric symptoms (e.g., depression, anxiety) and functioning (e.g., greater participation in community events) at post-treatment • IOK was reported to be helpful and acceptable
Purcell et al. (2018) *US*	VAMC	Veterans with PTSD who endorsed distress from killing or being responsible for the death of another in a war zone and completed IOK	Qualitative (interviews)	*Impact of killing* See Maguen et al. (2017)	• All participants reported that an intervention focused directly and explicitly on MI and killing was valuable • Participants identified the intervention’s self-forgiveness content as having the greatest benefit and reported that flexibility and structured assignments were positive aspects Many participants reported the intervention was too short, required more support and some expressed those benefits would be greater if the intervention were available closer to their return from deployment
Evans et al. (2021) *US*	Outpatient clinic	1 Male active-duty personnel with PTSD and exposure to PMIEs	Case study (from ongoing RCT)	*Massed prolonged exposure* (for PTSD) 15 individual sessions over 3 weeks (90 min) See Paul et al. (2014)	• Significant improvements in PTSD and functioning, with no changes found in depression symptoms at post-treatment Baseline CAPS: 43 Post-treatment CAPS: 35 Baseline PHQ: 19 Post-treatment PHQ: 20 Baseline functioning: 32 Post-treatment functioning: 22
Paul et al. (2014) *US*	VAMC	1 Male veteran with PTSD and exposure to PMIEs	Case study	*Prolonged Exposure* 9 weekly individual sessions *A cognitive-behavioral psychotherapy comprising psychoeducation, *in vivo* exposure, imaginal exposure, and processing*	•Reliable improvements in PTSD, depression and anxiety symptoms at post-treatment Baseline CAPS: 65 Post-treatment CAPS: 24 •No longer met PTSD diagnosis criteria at post-treatment
Snider (2015) *US*	Inpatient facility for PTSD	40 Active-duty personnel (85% male) with PTSD	Non-randomized trial	*Self-forgiveness: addressing MI* 4 weekly group sessions (90 min) *A brief psychotherapy designed to be adjunctive to Group CPT focusing on self-forgiveness* Comparator: Group CPT *Group variant of CPT*	• In the self-forgiveness group, significant improvements were reported for PTSD symptoms, self-forgiving feelings, and beliefs at post-treatment, with no significant differences found for shame or MI Baseline PCL-M: *M* = 63.70 (SD = 12.55) Post-treatment PCL-M: *M* = 56.10 (SD = 15.33) Baseline MIES: *M* = 35.25 (SD = 11.19) Post-treatment MIES: *M* = 34.40 (SD = 8.37) • In the Group CPT group, the only difference at post-treatment was on self-forgiving feelings and actions, with higher scores reported in the self-forgiveness group
Norman et al. (2014) *US*	VA hospital	10 Veterans (90% male) with guilt and distress related to trauma exposure	Pilot study	*Trauma informed guilt reduction therapy* 4–7 weekly individual sessions (90 min) *Transdiagnostic psychotherapy to address guilt, shame, and MI stemming from combat-related traumatic events over 4 modules*	• Statistically significant improvement found for PTSD symptoms at post-treatment, with 44% of veterans showing a clinically important improvement (10-point reduction in PTSD symptoms) Baseline CAPS: *M* = 81.4 (SD = 20.34) Post-treatment CAPS: *M* = 62.0 (SD = 36.5) • Non-significant medium to large improvements found for depression and guilt at post-treatment
Artra (2013) *US*	Not reported	8 Male veterans with PTSD	Pre/post	*Warrior’s journey retreat* 5 days *An expressive arts grieving retreat comprising guided introspection, body-based mindfulness, expressive arts, and group sharing*	• 7 of 8 participants reported a clinically significant change in PTSD symptoms at post-treatment. One participant reported a reliable change • Change in percentage in PTSD symptoms over 5 days ranged from 21% (8-point change) to 70% (42-point change)
Jones et al. (2020) *Canada*	Not reported	11 Veterans and active-duty personnel (91% male) with PTSD	Cross-over RCT (preliminary results)	*3MDR* (for PTSD) 6 weekly individual sessions (90 min) *3MDR is a virtual reality-based therapy administered on a treadmill and a synchronized virtual reality environment (with sound and visuals)* Comparator: Treatment as usual	• Statistically significant improvements in PTSD and MI symptom severity were found in the 3MDR group at post-treatment • Baseline CAPS: *M* = 46.8 (SD = 3.7) • Post-treatment CAPS: *M* = 14.4 (SD = 3.2) • Baseline MISS-M: *M* = 58.1 (SD = 12.9) • Post-treatment MISS-M: *M* = 52.27 (SD = 14.2)
Williamson et al. (2019) *UK*	Combat Stress	4 MHP (75% male) treating veterans exposed to PMIEs	Qualitative (interviews)	*Non-specific psychological approaches*	• Clinicians reported using a variety of adapted psychological treatment approaches (not specific to PTSD) to address PMIEs and associated feelings of guilt, shame, worthlessness (e.g., pie charts, compassion-focused therapy, and imagery re-scripting) • Poor understanding of MI among UK veteran clinical care teams, and limited number of treatment sessions were reported to be major challenges for effective treatment
Williamson et al. (2021) *UK*	NHS, Ministry of Defence	15 MHP (67% male) who had treating veterans or active-duty personnel exposed to PMIEs	Qualitative (interviews)	*Non-specific psychological approaches*	• No consensus between clinicians on the best treatment approach, with multiple treatments reportedly used in address MI-related distress (e.g., EMDR, compassion-focused therapy, elements of schema therapy, TF-CBT and mindfulness or an amalgamation of these approaches) • Clinicians reported 12–16 treatment session were required • Treatment challenges include maladaptive coping strategies, re-traumatization, confidentiality concerns and the need to build a trusting therapeutic relationship • Clinicians reported need for greater awareness of MI experience, impact, identification, and treatment options
*Chaplaincy delivered interventions*					
Ames et al. (2021) *US*	VAMC	2 Male veterans with PTSD and exposure to PMIEs	Case study (from ongoing RCT)	*Structured chaplain intervention* 12 weekly individual sessions (50 min) *Structured bio-psycho-social-spiritual forum for veterans to process trauma and MI using a spiritual perspective. Each session focuses on one dimension of MI (e.g., feeling betrayed, shame, loss of trust)*	Case #1 • Improvement in PTSD and MI symptoms (55% reduction in scores for PTSD and 19% for MI) Baseline PCL: 58 Post-treatment PCL: 26 Baseline MISS-M-SF: 66 Post-treatment MISS-M-SF: 55 Case #2 • Improvement in PTSD and MI symptoms (34% reduction in scores for PTSD and 25% for MI) Baseline PCL: 38 Post-treatment PCL: 25 Baseline MISS-M-SF: 52 Post-treatment MISS-M-SF: 47
Fleming (2020) *US*	Acute Psychiatric Care Unit	1 Male veteran with PTSD	Case study	*Warrior’s journey intervention* Single session *This narrative, meaning-making intervention is designed to improve motivation for treatment-seeking and help reduce post-trauma symptoms by following a universally shared spiritual metaphoric story of trauma recovery, with a focus on hope, meaning and guilt*	• Observed subjective improvement in the motivation for help-seeking and symptom relief
Pyne et al. (2021) *US*	VA PTSD clinic	13 Veterans (69% male) with PTSD	Pilot study	Mental Health Clinician Community Chaplain Collaboration (MC4) 6–12 individual weekly or biweekly session by phone or in person over a 3-month period *A flexible guide to spiritual counseling with a focus on forgiveness (self and others) and community reintegration*	• MC4 was generally feasible and acceptable, with 69% of veterans demonstrating acceptability of treatment (ERS score > 5) • There was minimal change across outcomes, however veterans who completed four or more sessions were four times as likely to experience improvement in symptoms than those who attended four or fewer sessions Baseline PCL: *M* = 50.5 (SD = 16.2) Post-treatment PCL: *M* = 48.1 (SD = 18.1) • Veterans reported the following aspects of MC4 as helpful: spiritual focus, emotional support, non-judgemental attitude, meeting face-to-face, sharing common combat experiences and spiritual beliefs with the facilitator. Of note, veterans reported that the short duration and lack of mental health training but a limitation of MC4
Chang et al. (2015) *US*	VA hospital	5 Chaplains (80% male) who provided spiritual care to veterans at the end of life with spiritual distress	Qualitative (interviews)	*Non-specific chaplaincy approaches*	Two treatment approaches were identified: • Religious approaches (e.g., religious scripts, confessing sins) • Non-religious approaches (e.g., recording military experience, meaning-making)
Drescher et al. (2018) *US*	National VA Chaplain Center	245 VA chaplains (85% male) who work with veterans exposed to PMIEs	Qualitative (survey)	*Non-specific chaplaincy approaches*	Various chaplain interventions were identified: • Pastoral/therapeutic presence (e.g., listening in a non-judgemental, compassionate way) • Therapeutic interventions (e.g., CT—reframing, challenging maladaptive thoughts and beliefs; narrative therapy, emotional processing, counselling, bibliotherapy, mindfulness, art therapy, rational emotive therapy, brief therapy) • Pastoral care (e.g., spiritual/religious counselling, prayer, and religious rituals) • Therapeutic exercises (e.g., psychoeducation, meditation/guided imagery, journaling) • Therapeutic process: Address self-evaluative conflict, promote meaning-making, Foster internal/external resources, Address emotion dysregulation
*Combined mental health practitioner and chaplaincy interventions*					
Harris et al. (2011) *US*	VAMC, community religious organization	54 Veterans (89% male) with trauma exposure (65% with PTSD)	RCT	*Building spiritual strength* 8 weekly group sessions (120 min) *Spiritually integrated, group counselling intervention designed to reduce symptoms of PTSD and promote psychospiritual development. It focuses on spiritual distress resolution using the veteran's existing meaning-making or faith orientation to help them address their traumatic experiences* Comparator: Waitlist	• In comparison to the waitlist group that showed no significant change in symptoms, veterans in the BSS group reported statistically significant reductions in PTSD symptoms at post-treatment • BSS Baseline PCL: *M* = 42.53 (SE = 4.21) Post-treatment PCL: *M* = 37.09 (SE = 3.99) • Waitlist Baseline PCL: *M* = 48.32 (SE = 3.70) Post-treatment PCL: *M* = 49.31 (SE = 3.00)
Harris et al. (2015) *US*	Local Catholic church	1 Male veteran with PTSD and depression	Case study	*Building spiritual strength* See Harris et al. (2011)	• Observed short-term symptom relief (reduced self-loathing) and improved motivation for help-seeking
Harris et al. (2018) *US*	VAMC	138 Veterans or active-duty personnel (76%) with PTSD	RCT	*Building spiritual strength* See Harris et al. (2011) Comparator:* Present Centered Group Therapy* 8 weekly group sessions (120 min) *Non-trauma focussed supportive counselling intervention*	• Both groups demonstrated clinically and statistically significant improvements in PTSD symptoms as measured by the CAPS at post-treatment • PTSD symptoms measured via self-report (PCL) did not indicate significant improvements • Veterans in BSS showed improvements in spiritual distress while those in PCGT reported increases in spiritual distress at post-treatment • BSS Baseline PCL: *M* = 58.51 (SD = 11.24) Post-treatment PCL: *M* = 53.24 (SD = 13.90) Baseline CAPS: *M* = 61.31 (SD = 21.02) Post-treatment CAPS: *M* = 37.70 (SD = 22.04) • PCGT Baseline PCL: *M* = 55.12 (SD = 10.83) Post-treatment PCL: *M* = 52.86 (SD = 13.47) Baseline CAPS: *M* = 61.12 (SD = 17.44) Post-treatment CAPS: *M* = 43.37 (SD = 22.87)
Antal et al. (2019) *US*	VA Mental Health Clinic	1 Male veteran exposed to PMIEs	Case study	*Moral Injury Group* 12 weekly group sessions (90 min) *Treatment consists of psychoeducation (e.g., MI, moral emotions) and a community ceremony consisting of music, ritual, spiritual discipline, and is an opportunity for veterans to testify about their MIEs and challenge the community to express responsibility*	Improvements were found in depression, religious struggles, self-compassion, and social functioning at post-treatment
Cenkner et al. (2021) *US*	VA Mental Health Clinic (outpatient)	40 Male veterans exposed to PMIEs	Pilot study	*Moral Injury Group* See Antal et al. (2019)	• Results indicate high engagement with a high rate of completion of MIG • Moderate improvements were found in depression (*ω*2 = 0.07) and psychological wellbeing (*ω*2 = 0.08), with small improvements found for religious struggles (*ω*2 = 0.03), post-traumatic growth (*ω*2 = 0.03) and self-compassion (*ω*2 = 0.05) at post-treatment • Concurrent treatment Baseline PHQ-9: *M* = 12.65 (SD = 5.84) Post-treatment PHQ-9: *M* = 9.00 (SD = 5.11) Baseline religious struggles: *M* = 58.80 (SD = 19.03) Post-treatment religious struggles: *M* = 53.00 (SD = 18.42) • Non-concurrent treatment Baseline PHQ-9: *M* = 12.18 (SD = 6.56) Post-treatment PHQ-9: *M* = 8.53 (SD = 5.35) Baseline religious struggles: *M* = 54.86 (SD = 22.39) Post-treatment religious struggles: *M* = 44.53 (SD = 18.49)
Starnino et al. (2019) *US*	VAMC	24 Treatment seeking veterans with PTSD	Uncontrolled study	*Search for Meaning Program* 8 weekly group sessions (90 min) *Mindfulness based psychoeducational and processing group focusing on spiritual, existential, and cognitive components to examine spiritual injury associated with combat-related PTSD*	• Significant, small to medium improvement in PTSD symptoms spiritual injury and negative religious coping were found at post-treatment • There was no significant improvement in positive religious coping Baseline PCL-5: *M* = 53.96 (SD = 11.90) Post-treatment PCL-5: *M* = 46.54 (SD = 17.33) Baseline SIS: *M* = 20.08 (SD = 4.19) Post-treatment SIS: *M* = 18.42 (SD = 4.41)
Starnino et al. (2019) Starnino et al. (2020) *US*	VA hospital	18 Veterans (94% male) with spiritual injury associated with combat-related PTSD completing the SFMP	Qualitative (interviews)	*Search for Meaning Program* See Starnino et al. (2019)	• Most participants reported shifts in meaning-making but most still unable to make sense of their PMIE • Connection to others with similar experiences was viewed as positive by most participants

*ACT* acceptance and commitment therapy; *AD* adaptive disclosure; *AUD* alcohol use disorder; *AUDIT* alcohol use disorders identification test; *BDI* beck depression inventory; *BSS* building spiritual strength; *CAPS* Clinician Administered PTSD Scale; *CBT* cognitive-behavioral therapy; *CPT* cognitive processing therapy; *CPT-C* cognitive processing therapy—cognitive version; *CT* cognitive therapy; *EBP* evidence-based psychotherapy; *IOK* impact of killing; *M* mean; *MC4* Mental Health Clinician Community Chaplain Collaboration; *MHP* mental health practitioner; *MI* moral injury; *MIAS* Moral Injury Appraisals Scale; *MIEs* morally injurious experiences; *MIES* Moral Injury Event Scale; *MIG* Moral Injury Group; *MISS-M* Moral Injury Symptom Scale Military; *MISS-M-SF* Moral Injury Symptom Scale-Military Version Short Form; *NL* Netherlands; *PCGT* Present Centered Group Therapy; *PCL* post-traumatic checklist; *PE* prolonged exposure; *PHQ* Patient Health Questionnaire; *PMIEs* potentially morally injurious events; *PTCI* post-traumatic cognitions inventory; *PTSD* post-traumatic stress disorder; *RCT* randomized control trial; *SD* standard deviation; *SIS* Spiritual Injury Scale; *TF-CBT* trauma-focused CBT; *UK* United Kingdom; *US* United States; *VA* veteran affairs; *VAMC* veteran affairs medical center;* WSAS* Work and Social Adjustment Scale

### Study Characteristics

A total of 26 quantitative studies were identified consisting of 11 case studies, 8 pre-post studies, 6 randomized control trials (RCTs), and a single program evaluation using pre and post measures. Most studies (*k* = 23, 88%) originated from the United States, with single studies from Canada, United Kingdom and the Netherlands. Studies were mostly conducted with military samples (*k* = 24, 92%) and involved male personnel (88% of studies comprised 70% or more male personnel). Most studies examined interventions delivered by MHPs (65%, *k* = 17), three studies (12%) examined interventions delivered by chaplains, and six studies (23%) examined interventions co-delivered by MHP and chaplains (see Table 1). Most studies assessed changes in PTSD (k = 22, 85%) and depression scores (*k* = 13, 50%) to determine intervention outcome. A smaller number of studies (*k* = 8, 31%) assessed MI as an outcome, variously using the Moral Injury Events Scale (*k* = 3), the Moral Injury Symptom Scale—Military (*k* = 2), the Moral Injury Attributions Scale (*k* = 1), the Expressions of Moral Injury Scale (*k* = 1) and Cognitive Fusion Questionnaire-Moral Injury (*k* = 1). There were five studies (19%) that assessed religious struggles or spiritual distress as an outcome.

A total of nine qualitative studies were identified. Most qualitative studies (*k* = 7, 78%) originated from the United States, with two studies from the United Kingdom (22%). All qualitative studies were conducted within a military setting, including mental healthcare service settings (e.g., a VA Medical Center, *k* = 9, 89%), with a single study at a national VA Chaplain Center. Most qualitative studies included military personnel (veterans or active-duty personnel) completing psychotherapies for PTSD or MI (*k* = 4, 44%), with a smaller proportion including mental health practitioners working with veterans with PTSD and PMIE exposures (*k* = 3, 33%), or chaplains providing care to veterans with MI or spiritual distress (*k* = 2; 22%) (see Table 1).

### Study Themes

Using a narrative descriptive synthesis approach, six key themes emerged: 1) Mental health practitioner approaches to MI; 2) Interventions for PTSD; 3) Adjunctive and alternative interventions for MI; 4) Non-psychological interventions for MI; 5) Chaplaincy approaches to MI; and 6) Chaplains and mental health practitioners combined approaches to MI. Each of these are further discussed below.

### MHP Approaches to MI

There were two qualitative studies that examined MHP approaches to supporting military personnel with MI-related mental health problems. Several different approaches were reported, with no consensus between clinicians on the best intervention approach (Williamson et al., 2019, 2021).

Most of the quantitative studies identified in the search (17 of 26) assessed interventions facilitated by MHPs to address MI. These included: (1) interventions for PTSD using standard evidence-based PTSD interventions (PE, CPT; five studies), adapted PTSD interventions (CPT, Cognitive Therapy, Brief Eclectic Psychotherapy; three studies), or emerging PTSD interventions (Multi-modular Motion-assisted Memory Desensitization and Reconsolidation, Trauma Informed Guilt Reduction; two studies); (2) interventions targeting MI as an adjunct to standard intervention for PTSD (Impact of Killing, Self-forgiveness: Addressing MI; two studies) or as alternatives to standard intervention for PTSD (Adaptive Disclosure, Acceptance and Commitment Therapy; four studies); and (3) a non-psychological intervention (Warrior Journey Retreat; one study). Details of the studies are provided in the following section.

### Interventions for PTSD

#### Standard Evidence-Based PTSD Interventions

A number of studies have investigated the efficacy of standard evidence-based PTSD interventions for the treatment of MI. Two of these standard PTSD intervention efficacy studies compared mental health and wellbeing symptom reduction in those who had experienced traditional PTSD-related traumas such as fear and/or loss in combat, compared to those who had experienced MI-related index traumas such as causing or witnessing civilian casualties.

Two case studies found reductions in PTSD and depression symptoms for US veterans with MI-related injurious index traumas following standard PE and CPT (Held et al., 2018; Paul et al., 2014). One study assessed the effectiveness of an intensive outpatient PTSD program (massed-CPT), consisting of group CPT and individual CPT (in conjunction with mindfulness and yoga sessions) and found similar trajectories for PTSD and depression symptoms in veterans endorsing MI-related PTSD (e.g., history of MI; index trauma involved a PMIE) compared to those with PTSD without a PMIE exposure (Held et al., 2021). Another case study found reductions in PTSD but no change in depression symptoms, following massed-PE in US active-duty personnel with MI-related index traumas (Evans et al., 2021). Finally, a secondary analysis of an RCT comparing individual and group CPT (without the written accounts) identified no difference in symptoms of PTSD, depression, anxiety or suicide ideation in US active-duty personnel endorsing a MI-related index trauma (committing, witnessing or being the victim of acts perceived to be gross violations of moral or ethical standards) compared to those with other trauma types (e.g., exposure to threat of death of self or others, aftermath of violence, or traumatic loss) (Hawkins, 2021).

Of interest, a qualitative study of veterans with exposure to a PMIE who had completed PE or CPT, reported that MI was not identified or sufficiently discussed during therapy and that the intervention had little impact on MI during or after intervention (e.g., did not target guilt or shame, had no long lasting impact on functioning) (Borges et al., 2020).

#### PTSD Interventions Adapted for MI

The literature review identified three PTSD interventions that have been adapted specifically to address MI: CPT, Cognitive Therapy and Brief Eclectic Psychotherapy. CPT for PTSD has been adapted in a 12-session Spiritually Integrated CPT (SI-CPT) intervention. SI-CPT focuses on challenging maladaptive thinking patterns through gradual exposure, cognitive processing and cognitive restructuring. To accommodate the potential for appropriate or legitimate self-blame or guilt following a PMIE, cognitive restructuring within SI-CPT challenges interpretations of trauma that serve to maintain distress, using spiritual resources (e.g., mercy, repentance, forgiveness, spiritual surrender, prayer/contemplation, divine justice, hope, and divine affirmations), and rituals (e.g., confession, penance). Quantitative evidence for SI-CPT is limited to a single case study (Pearce et al., 2018), which demonstrated improvements in PTSD symptoms for a veteran with PTSD associated with a PMIE.

Cognitive Therapy has also been examined as a potential intervention approach to address MI in the context of PTSD (CT-PTSD Addressing MI; Murray & Ehlers, 2021). A single case study of a British doctor with MI-related PTSD reported improvements in symptoms of PTSD, depression and functioning following CT-PTSD. The intervention involved psychoeducation on MI, incorporating reclaiming values and self-identity, discussing shame and guilt prior to accessing the traumatic memory, identifying and addressing distorted or over-generalized appraisals within the trauma memory, and acceptance and moving on (e.g., acts of reparation).

Lastly, Brief Eclectic Psychotherapy for Moral Trauma is a 16-session individual cognitive-behavioral, psychodynamic, constructivist, and systemic psychotherapy including psychoeducation, gradual imagery exposure to the moral trauma, mementos and writing tasks, and processing of thoughts and emotions (including shame and guilt). Evidence to date is restricted to a single case study of a male refugee with PTSD and history of military service in his home country. The case study reported improvements in PTSD and MI appraisals after completing the intervention (de la Rie et al., 2021).

#### Emerging PTSD Interventions Adapted for MI

Emerging PTSD interventions that have been used to address MI include Trauma Informed Guilt Reduction (TrIGR) and Multi-modular Motion-assisted Memory Desensitization and Reconsolidation (3MDR). TrIGR is a 4–7-session transdiagnostic psychotherapy used to address guilt, shame, and MI stemming from combat-related traumatic experiences. Patients are encouraged to accurately appraise their trauma-related guilt, taking into account the full context in which the event occurred and taking steps towards leading a meaningful life through re-engaging with their values. A pilot study of TrIGR in 10 US veterans (Norman et al., 2014) found clinically significant improvements in PTSD symptoms. Finally, 3MDR, an emerging virtual reality-based therapy for PTSD, has also been found to significantly reduce PTSD and MI symptom severity based on a crossover RCT of 40 Canadian veterans and active-duty personnel (Jones et al., 2020).

### Adjunctive and Alternative Psychotherapies for MI

Another body of research highlights interventions that have been specifically developed to address MI. These include two interventions intended as an adjunct to standard interventions for PTSD (Impact of Killing and Self-forgiveness: Addressing MI) and two alternative interventions (Active Disclosure and Acceptance and Commitment Therapy).

Impact of Killing is a 6–8 session, cognitive-behavioral intervention that has an explicit focus on the act of killing, as a PMIE involving transgression of personal or shared morals and leading to MI (Burkman et al., 2021). Empirical evidence to date assessing Impact of Killing is limited to a pilot RCT of 35 veterans who had previously completed standard treatment for PTSD (Maguen et al., 2017), which found the intervention to be both helpful and acceptable. In addition, veterans in the Impact of Killing condition reported statistically significant greater improvements in PTSD symptoms, general psychiatric symptoms (e.g., depression, anxiety) and functioning (e.g., greater participation in community events) relative to the wait-list control condition. Qualitative evidence has supported the acceptability and feasibility of the Impact of Killing in a veteran sample (Purcell et al., 2018) and a sample of mental health practitioners (Burkman et al., 2019). Further, these studies found that the intervention addressed a clinical need that was not met by other standard interventions for PTSD.

Self-forgiveness: Addressing MI is a brief 4-session intervention that involves: (1) psychoeducation on forgiveness, shame, and guilt; (2) the identification of violated values, moral and beliefs; and (3) written accounts of trauma exposure. A non-randomized trial comparing group CPT and group CPT plus the self-forgiveness modules (Snider, 2015) indicated no significant difference between the two conditions on PTSD as an outcome, with both showing significant reductions in PTSD symptoms. However, veterans completing the self-forgiveness modules reported significantly higher feelings and actions related to self-forgiveness in comparison to the veterans completing group CPT only.

Adaptive Disclosure has been proposed as an alternative intervention for MI. Adaptive Disclosure is an emotion-focused, cognitive-behavioral therapy with 6- and 8-session protocols developed specifically for active-duty military personnel (Litz et al., 2021). The intervention is designed to flexibly address MI, traumatic loss and grief, or the impacts of life-threatening experiences. It involves psychoeducation, imaginal exposure, and experiential processing. In comparison to standard PE, the exposure components of Adaptive Disclosure focus on identifying distressing appraisals and cognitions rather than fear extinction, and standard cognitive restructuring exercises are largely replaced with experiential exercises (Litz et al., 2021). Adaptive Disclosure has been tested in a pilot open trial (Gray et al., 2012) and a non-inferiority RCT (Litz et al., 2021). In the preliminary open trial of 44 active-duty US military personnel, significant improvements were found for PTSD symptoms, depression symptoms, post-traumatic cognitions and post-traumatic growth (Gray et al., 2012). Results from the non-inferiority RCT with 122 active-duty US military personnel, evidenced reductions in PTSD symptoms in the Adaptive Disclosure condition to be comparable to reductions from CPT—Cognitive Therapy version.

Acceptance and Commitment Therapy (ACT) is a transdiagnostic intervention that has been proposed as a promising intervention for MI. ACT can be delivered individually or in group, in person or via telehealth, and over 8 or 12 sessions. The intervention focuses on openness, awareness and engagement to (1) foster psychological and behavioral flexibility; (2) promote acceptance of experiences; and (3) promote commitment to actions toward value-based behavior (Walser & Wharton, 2021). Evidence from a pre-post study of 33 US veteran participants receiving ACT in a group setting indicated a favorable response with statistically significant reductions in symptoms of PTSD and depression, as well as enhanced self-reported wellbeing. Unfortunately, intervention effects were not maintained to follow-up (Bluett, 2017). Evidence from a case study of ACT delivered via telehealth also indicated a favorable response with statistically significant reductions in symptoms of PTSD and depression. (Borges, 2019). In addition, 11 US veterans in a PTSD residential treatment program reported benefits from group ACT intervention, and indicated that they would recommend ACT to other veterans (Farnsworth et al., 2017).

### Non-psychological Interventions for Moral Injury

Warrior Journey Retreat is a 5-day grieving retreat involving guided introspection, body-based mindfulness, expressive arts and group sharing. Evidence from a single pre-post study reported clinically significant improvements in PTSD symptoms in seven of eight US veterans who completed the Warrior Journey Retreat (Artra, 2013).

### Chaplaincy Approaches to MI

The literature search identified two qualitative studies assessing chaplain’s experiences of providing spiritual care to veterans with MI. The studies explored what support was provided, and how effective chaplains’ approaches were in helping veterans address spiritual distress. Chaplains reported using both religious and non-religious approaches, comprising pastoral care (e.g., spiritual counseling, religious scripts, listening), and therapeutic processes (e.g., psychoeducation, meditation and mindfulness, emotional processing, narrative therapy) (Chang et al., 2015; Drescher et al., 2018). Chaplains reported success in helping veterans with spiritual distress, particularly when the distress stemmed from religion, but also acknowledged the need for referrals to mental health practitioners to address the psychological aspects of spiritual distress (Chang et al., 2015).

There were also three quantitative studies of chaplaincy approaches used to address MI. The Mental Health Clinician Community Chaplain Collaboration (MC4) is a 6–12-week, individual, spiritual-based counseling intervention that focuses on forgiveness of a higher power, of others and of self, as well as community reintegration through both community connection and community service to make amends. Evidence from a feasibility study with 13 US veterans found MC4 to be feasible and acceptable to participants (Pyne et al., 2021), however there were minimal improvements in PTSD symptoms, psychological distress, self-forgiveness, guilt and shame. Veterans reported the following components of MC4 as beneficial: spiritual focus of the intervention (92%); emotional support (76%); non-judgmental manner of facilitators (i.e., pastors, chaplains, or community clergy) (69%); shared combat experience (46%) and spiritual beliefs (38%) of facilitators. Approximately one-third of participating veterans reported the lack of mental health training of facilitators as a limitation of MC4.

The Structured Chaplain Intervention is a 12-session, structured bio-psycho-social-spiritual intervention enabling veterans to process trauma using a spiritual lens. Topics include dimensions of MI, such as feeling betrayed, guilt, shame, moral concerns, religious struggles, loss of religious faith/hope, loss of trust, loss of meaning/purpose, difficulty forgiving and self-condemnation. A case study with two US veterans participating in an ongoing RCT of the Structured Chaplain Intervention provided preliminary low certainty evidence suggesting improvements in symptoms of PTSD as well as MI (Ames et al., 2021).

The Warrior’s Journey Intervention (unrelated to the Warrior Journey Retreat mentioned above) is a narrative meaning-making intervention, intended to motivate treatment seeking in veterans experiencing symptoms of PTSD. Chaplains share a spiritual, metaphoric adventurous story of trauma recovery, designed to impact on hope, meaning and guilt. A single case study (Fleming, 2020) provided low certainty evidence suggesting improved motivation for help-seeking and symptom relief in a veteran who completed the single session Warrior’s Journey Intervention.

### MHP and Chaplains—Combined Approaches to MI

The literature search identified three interventions that combined psychological and chaplaincy approaches to addressing MI and these were assessed in six studies. Building Spiritual Strength (BSS) is an eight-session group-based counseling intervention specifically designed to reduce symptoms of PTSD and promote psychospiritual development by encouraging participants to actively address their MI-related distress using pre-existing spiritual resources (Usset et al., 2021). The components of BSS include reframing, discussing evil in the world, and using meditation and prayer as an active coping strategy. Evidence for the effectiveness of BSS in addressing MI symptoms comes from a case study (Harris et al., 2015) and two RCTs (Harris et al., 2011, 2018). The case study of a US veteran with PTSD and depression indicated short-term symptom relief (e.g., reduced self-loathing) and improved motivation for help-seeking (Harris et al., 2015). The first RCT involving 54 US veterans (Harris et al., 2011), reported statistically significant reductions in PTSD symptoms at postintervention in veterans in the BSS group relative to those in the wait-list control. The second RCT of 138 US veterans and active-duty personnel (Harris et al., 2018) used Present-centered Group Therapy as a control condition. The authors found that both groups demonstrated a clinically and statistically significant improvement in PTSD symptoms; however, participants in the BSS condition showed improvements in spiritual distress while those who received Present-centered Group Therapy reported increases in spiritual distress.

Moral Injury Group, is a 12-session intervention incorporating psychoeducation focused on components of morality (i.e., MI, moral emotions, moral values, moral dilemmas, and moral disengagement) and spiritual practices integrated within the Community Ceremony, and consisting of music, ritual and spiritual discipline. The Community Ceremony offers an opportunity for Veterans to testify about their MI experience and challenge the community to ‘express responsibility’ for those sent to war on their behalf, through ceremonial ritual such as lighting “Candles of Lament" (visible reminders of people who died in warfare), the Reconciliation Circle (veterans surround non-veterans in a symbolic circle of protection, followed by non-veterans surrounding veterans, symbolic of ‘having the backs’ of veterans), a water cleansing ritual (handwashing and prayer), and Candles of Hope (candles are taken by non-veterans as a symbol of shared responsibility). A single case study of a US veteran found improvements in depression, religious struggles, self-compassion, and social functioning (Antal et al., 2019). Evidence from a proof-of-concept study with 40 veterans indicated high rates of completion, with small to moderate improvements in depression, psychological wellbeing, religious struggles, post-traumatic growth and self-compassion at postintervention. (Cenkner et al., 2021).

The Search for Meaning Program has been implemented within a Veteran Affairs (VA) Medical Center in the USA since 2012 (Starnino et al., 2019). The Search for Meaning Program is an 8-session group intervention involving spirituality- and mindfulness-based PTSD psychoeducation. Topics include spiritual wounding and subsequent emotions (e.g., anger, sadness, betrayal, hopelessness), impact of trauma on core beliefs, avoidance, meaning-making processes, strategies to address anger and grief, and forgiveness. A preliminary evaluation of 24 US veterans completing the Search for Meaning Program at the VA Medical Center found significant improvements in PTSD symptoms, spiritual injury and negative religious coping (Starnino et al., 2019). A qualitative study involving interviews with 18 US veterans following completion of the Search for Meaning Program reported that the veterans found the intervention to be helpful but not sufficient to enable them to make meaning from their PMIE (Starnino et al., 2019, 2020).

## Discussion

This scoping review provides an overview of the literature describing the approaches taken by MHPs and chaplains to address MI, as well as a summary of the published evidence to date regarding the effectiveness of each approach. Despite rapid growth in interest in the construct of MI and exponential growth in the literature describing the experience of MI within different populations, there are still very few RCTs (*k* = 6) or pre-post studies (*k* = 8), that have been designed to assess the effectiveness of interventions for MI.

The emerging evidence includes studies that have investigated a variety of different approaches to MI designed to be delivered by MHPs. These include standard PTSD interventions (PE and CPT), an adjunctive intervention (Impact of Killing) and two alternative interventions (Adaptive Disclosure and Acceptance and Commitment Therapy).

While several studies found PE and CPT to be acceptable, feasible and effective for symptoms of PTSD in those with MI, a qualitative study found that the interventions had limited impact on MI-related symptoms, and for some participants, benefits were not maintained (Borges et al., 2020). As a result, individuals with a history of PMIE exposure, who receive standard PTSD interventions may not experience any sustained reduction in distress associated with MI-related symptoms such as shame, loss of trust, and guilt. This possibility is theoretically congruent as violation of one’s morals or ethics is not typically addressed within standard TF-CBT. Relatedly, event reappraisals, a component of standard TF-CBT PTSD interventions, may be less relevant for MI wherein the accurate appraisal is a causal mechanism of distress. Interventions that promote self-forgiveness and forgiveness of others may be more effective in addressing MI (Litz et al., 2009; Maguen et al., 2017; Steinmetz & Gray, 2015).

There is emerging evidence for MHP-delivered interventions that are designed to be delivered as an adjunct to standard PTSD interventions and specifically target symptoms associated with exposure to PMIEs. The feasibility, acceptability and potential effectiveness of Impact of Killing as an adjunctive therapy for MI was supported by a small pilot study (Maguen et al., 2017) and two qualitative studies (Burkman et al., 2019; Purcell et al., 2018). Evidence from uncontrolled studies suggested that Acceptance and Commitment Therapy may reduce PTSD and depression symptoms as well as enhance general wellbeing in individuals who had been exposed to a PMIE; however results from a rigorous longitudinal study indicated that benefits in MI-related outcomes were not sustained and in fact some symptoms *increased* from baseline to one-month post intervention Bluett (2017), thus additional longitudinal trials – ideally RCTs—are needed. Finally, Adaptive Disclosure has been found in an open pilot trial (Gray et al., 2012) and a non-inferiority trial against CPT-C (Litz et al., 2021) to improve PTSD and depression symptoms in active-duty military personnel with PTSD. There was no long-term follow up data in either study, so the sustainability of intervention effects remain unknown.

Other MHP-delivered interventions with less evidence to date include Brief Eclectic Psychotherapy for Moral Trauma, Cognitive Therapy, Trauma Informed Guilt Reduction, Self-forgiveness: Addressing MI, Multi-modular Motion-assisted Memory Desensitization and Reconsolidation, and the Warriors Journey Retreat. Studies assessing the effectiveness of these interventions varied in methodological rigor and more research is required before we can be confident in their effectiveness.

The scoping review identified two chaplain-delivered interventions for which evaluations have been published, the Structured Chaplain Intervention and the Mental Health Clinician Community Chaplain Collaboration. However, both evaluations were based on case studies, providing low certainty evidence. The paucity of evidence for chaplaincy approaches to MI does not indicate that chaplains are not addressing MI. A large qualitative study of 245 US Veterans Affairs chaplains reported that chaplains working with veterans exposed to PMIEs draw on a broad range of therapeutic interventions including religious/spiritual-based approaches and psychotherapeutic approaches (Drescher et al., 2018). The potential for combined psychological and chaplaincy approaches has been showcased in the combined approaches described in this review with initial evidence of small to moderate intervention effects. There is, however, considerable room for further development and testing of combined approaches to addressing MI.

One of the most obvious shortcomings in the MI intervention literature included in the current scoping review, is the absence of comprehensive and validated change measurements specific to diverse MI outcomes. MI involves broad psychosocial and spiritual outcomes, including emotions (e.g., guilt, shame, anger), self-perception, interpersonal functioning and spiritual/existential beliefs (Yeterian et al., 2019). Yet most of the studies included in the review relied on changes in PTSD and depression symptoms to assess the effectiveness of MI interventions. A small number of studies assessed MI outcomes using three different MI measures: Moral Injury Appraisals Scale, which focuses on cognitive appraisal of PMI experiences; Moral Injury Event Scale (MIES), which is intended to measure PMIE exposures rather than MI outcomes; and Moral Injury Symptom Scale Military which measures some but not all MI outcomes. Unfortunately, none of these measures were developed using best practice test construction and validation approaches, relying instead upon the compilation of items from existing scales and informed assumptions regarding the nature of MI symptoms (Yeterian et al., 2019). A validated measure of MI outcomes that captures the full spectrum of MI across psychosocial and spiritual domains developed following best practice methodology including input from the target population and designed to assess MI symptoms in a broader population than just military personnel or veterans is needed to properly assess the impacts of psychological and chaplaincy approaches to MI and whether there is incremental utility in a combined approach. The routine use of such a measure, alongside measures of PTSD and depression symptoms, would provide a more comprehensive understanding of outcomes from interventions for MI.

### Limitations

The focus of this scoping review was peer-reviewed literature within 11 electronic databases listing psychological and/or chaplaincy approaches to support adults who have experienced MI or been exposed to PMIEs. While credible psychological literature can be identified within such databases, chaplaincy and spiritual care literature within books and book chapters regarding intervention programs for MI/PMIEs were not included. Therefore, this review did not consider the efficacy of any social or community intervention programs run and published by ecclesiastical or other religious organizations. Also, while some programs were noted within the peer-reviewed literature to be currently under the process of development or validation, these were not included in the current review given their ongoing development and evaluation.

### Conclusion

This scoping review, based on a systematic search of the research literature, provides a comprehensive summary of current evidence for interventions to address MI outcomes and related symptoms. Unfortunately, most studies used symptoms of PTSD or depression as key outcome measures, pointing to the urgent need for a widely accepted, psychometrically sound measure of MI. More broadly, the quality and depth of research to date does not allow conclusions to be drawn about preferred approaches, much less of the relative benefits of psychological, chaplaincy, and combined psychological and chaplaincy approaches to addressing MI. The psychosocial and spiritual impacts of MI, suggest that a combined approach may be optimal; however, there is insufficient evidence to date to support such a conclusion. Future research should prioritize the development and testing of a multidisciplinary psychosocial spiritual model of care for MI.

## Appendix 1: Example Search Strategy Conducted in Medline Database on 9th July 2021

**Table:**

Step	Search terms	No. of records
1	Moral injur*.mp	318
2	Spiritual injur*.mp	11
3	Morally injurious.mp	78
4	Moral distress.mp	1221
5	Spiritual distress.mp	323
6	Moral dissonance.mp	3
7	Spiritual dissonance.mp	2
8	Moral conscience.mp	42
9	((Trauma or traumatic) adj6 (ethic* or belief* or believing or moral*)).mp	618
10	Betrayal.mp	585
11	1 or 2 or 3 or 4 or 5 or 6 or 7 or 8 or 9 or 10	2971
12	Chaplain*.mp	2340
13	Padre*.mp	1279
14	Madre*.mp	2162
15	Imam*.mp	1811
16	Minister.mp	3105
17	Ministers.mp	1449
18	(Monk or monks).mp	731
19	(Pastor or pastors or pastoral).mp	6201
20	(Rabbi or rabbis).mp	227
21	Spiritual care.mp	1973
22	Intervention*.mp	1,149,192
23	(Treatment* or therap* or psychotherap*).mp	8,977,168
24	Support.ti,kf,ab	1,077,507
25	(Counseling or counselling).mp	127,895
26	Psychologist*.mp	16,669
27	Psychiatrist*.mp	26,490
28	Mental health provider*.mp	1486
29	Mental health professional*.mp	6175
30	Mental health practitioner*.mp	774
31	Mental health therapist*.mp	63
32	12 or 13 or 14 or 15 or 16 or 17 or 18 or 19 or 20 or 21 or 22 or 23 or 24 or 25 or 26 or 27 or 28 or 29 or 30 or 31	10,241,374
33	11 and 32	1571
34	Limit 33 to english language	1529
