# Correction to: Moral Injury, Chaplaincy and Mental Health Provider Approaches to Treatment: A Scoping Review

**DOI:** 10.1007/s10943-022-01560-2

**Published:** 2022-04-16

**Authors:** Kimberley A. Jones, Isabella Freijah, Lindsay Carey, R. Nicholas Carleton, Peter Devenish‑Meares, Lisa Dell, Sara Rodrigues, Kelsey Madden, Lucinda Johnson, Fardous Hosseiny, Andrea J. Phelps

**Affiliations:** 1 Phoenix Australia - Centre for Posttraumatic Mental Health, Department of Psychiatry, University of Melbourne, Carlton, VIC 3053 Australia; 2 Department of Public Health, La Trobe University, Melbourne, Australia; 3 Department of Psychology, University of Regina, Regina, Canada; 4 Graduate School of Business, Queensland University of Technology, Brisbane, Australia; 5 Canadian Centre of Excellence On Post-Traumatic Stress Disorder (PTSD) and Related Mental Health Conditions, Ottawa, Canada

## Correction to: Journal of Religion and Health (2022) 61:1051–1094 https://doi.org/10.1007/s10943-022–01534-4

In this article, the affiliation details for the author “Fardous Hosseiny” were incorrectly given as “Department of Public Health, La Trobe University, Melbourne, Australia” but should have been “Canadian Centre of Excellence on Post-Traumatic Stress Disorder (PTSD) and Related Mental Health Conditions, Ottawa, Canada”. This has been corrected with this erratum.

